# Exploring Combinations of Dual Functionality, Education, and Mortality: New Findings from MIDUS

**DOI:** 10.1093/geroni/igaf122.2315

**Published:** 2025-12-31

**Authors:** Jonghwa Do, Jieun Song, Carol Ryff

**Affiliations:** University of Wisconsin–Madison, Madison, Wisconsin, United States; University of Wisconsin–Madison, Madison, Wisconsin, United States; University of Wisconsin–Madison, Madison, Wisconsin, United States

## Abstract

There has been growing attention on the role of functionality in older adults, as diseases become less lethal and more chronic over time. While lower cognition and poorer physical health are well-established predictors of mortality risk, less is known about their combined effects. This study examines mortality risks across dual functionality, assessed by cognition and physical functioning, among adults aged 60 and older using data from Midlife in the U.S. (MIDUS) (N = 1,578). Cognition and physical functioning were assessed at MIDUS III (2013-2014) via Brief Test of Adult Cognition by Telephone (BTACT) and SF-36. Mortality data were obtained via National Death Index 2023 files. Dual functionality groups were identified based on quartiles of cognitive and physical functioning: Both High (n = 245), Both Mid (n = 445), Both Low (n = 494), Cognition High–Physical Low (n = 170), and Cognition Low–Physical High (n = 224). Results from Cox proportional hazard models showed that imbalance between cognitive and physical functionality predicted greater mortality risks, compared to Both High or Both Mid functioning. Specifically, mortality risks were significantly greater in Cognition High–Physical Low (HR = 3.06; 95% CI = 1.52–6.16) and Cognition Low–Physical High (HR = 3.91; 95% CI = 1.95–7.85) groups, compared with Both High group, and somewhat comparable with Both Mid group (HR = 2.59; 95% CI = 1.35-4.97). Further, the detrimental effects of imbalanced functionality on mortality risks were observed regardless of educational attainment. The findings suggest substantial impacts of imbalanced functionality on mortality risks. It warrants future studies to examine the pathways through which imbalanced functionality increases mortality, which can shed light on possible interventions.

